# Exploration of Computational Approaches to Predict the Toxicity of Chemical Mixtures

**DOI:** 10.3390/toxics7010015

**Published:** 2019-03-19

**Authors:** Supratik Kar, Jerzy Leszczynski

**Affiliations:** Interdisciplinary Center for Nanotoxicity, Department of Chemistry, Physics and Atmospheric Sciences, Jackson State University, Jackson, MS 39217, USA; supratik.kar@icnanotox.org

**Keywords:** computational, in silico, mixture, QSAR, toxicity

## Abstract

Industrial advances have led to generation of multi-component chemicals, materials and pharmaceuticals which are directly or indirectly affecting the environment. Although toxicity data are available for individual chemicals, generally there is no toxicity data of chemical mixtures. Most importantly, the nature of toxicity of these studied mixtures is completely different to the single components, which makes the toxicity evaluation of mixtures more critical and challenging. Interactions of individual chemicals in a mixture can result in multifaceted and considerable deviations in the apparent properties of its ingredients. It results in synergistic or antagonistic effects as opposed to the ideal case of additive behavior i.e., concentration addition (CA) and independent action (IA). The CA and IA are leading models for the assessment of joint activity supported by pharmacology literature. Animal models for toxicity testing are time- and money-consuming as well as unethical. Thus, computational approaches are already proven efficient alternatives for assessing the toxicity of chemicals by regulatory authorities followed by industries. In silico methods are capable of predicting toxicity, prioritizing chemicals, identifying risk and assessing, followed by managing, the risk. In many cases, the mechanism behind the toxicity from species to species can be understood by in silico methods. Until today most of the computational approaches have been employed for single chemical’s toxicity. Thus, only a handful of works in the literature and methods are available for a mixture’s toxicity prediction employing computational or in silico approaches. Therefore, the present review explains the importance of evaluation of a mixture’s toxicity, the role of computational approaches to assess the toxicity, followed by types of in silico methods. Additionally, successful application of in silico tools in a mixture’s toxicity predictions is explained in detail. Finally, future avenues towards the role and application of computational approaches in a mixture’s toxicity are discussed.

## 1. Introduction

Exploration of chemicals’ toxicity towards living things as well as ecosystems should be one of the primary steps before introducing into industry or commerce any new chemicals and/or drugs. Environmental hazards are the result of toxic chemicals and the majority of them are due to complex chemical mixtures [1]. The majority of industries only provide single chemical toxicity data during the introduction of specific chemicals. Thus, multi-chemical mixture toxicity data is rarely available and most interestingly, with a different ratio of the same chemicals, mixtures may show different toxicity responses. The ecotoxicity of mixtures can be highly reliant on the shape of the spread of its individual components. The existence of highly toxic compounds is not essentially reflected in the computed average structure of a mixture. Thus, for chemicals one cannot expect reasonable mathematical association between the assessed toxicity and the molecular descriptors of the structure, which is in most cases efficiently accomplished for single chemicals [2].

Unlike single chemicals, mixtures have no or very few experimental datasets for toxicity. The reasons mentioned below make database preparation work difficult and multifaceted:

(a) toxicity data vary with different combinations of the same chemicals in a mixture;
(b) form of exposure;
(c) identification of each chemicals in a specific mixture is also difficult due to the presence of very small quantities; and
(d) complex interactions among chemicals.

Thus, first and foremost the requirement is to accumulate the available experimental toxicity data of mixtures for diverse species and different compartments of the environment. These data are the input parameters for computational study and the first source for filling data gaps [3,4].

Animal study and in vitro models are expensive and require a long time for toxicity testing. In addition, ethical considerations about killing animals is another important factor. Thus, computational approaches for assessing the toxicity of chemicals are considered valuable. Although the majority of computational approaches have been implemented for toxicity modelling of single chemicals, they can be also employed tactfully for mixtures. In silico methods may help in risk assessment to examine, simulate, visualize and predict the toxicity of chemicals economically in a short time, without the sacrifice of animals. In silico toxicology objectives complement prevailing toxicity tests to predict toxicity, prioritize chemicals, and guide toxicity tests. In silico methods like classical quantitative structure-activity relationship (QSAR) and machine learning (ML) approaches trained on experimental data could be beneficial to make predictions on the probable toxicity of mixtures. The QSAR and ML models are capable of modelling the relationships between toxicity response and properties based on chemical structure as well as physicochemical properties. The relationships obtained can later be reliably used for a new or untested mixture’s toxicity prediction [5]. 

A good number of computational models had been developed employing the QSAR approach [6,7,8,9,10]. However, approaches like read-across, docking, and structural alerts need to be used to make the computational model-based toxicity prediction more efficient and reliable one. Additionally, the data gap filling and making of regulatory guideline are two major factors for the necessity of application computational approaches in toxicity prediction of mixture. The presented review analyses the reasons and hypothesis for the assessment of mixture toxicity followed by discussion of types of in silico methods and already developed successful computational models. 

## 2. Why Exploration of Toxicity of Chemical Mixtures is Important?

Single compound chemical toxicity in the environment is a myth as most of the time chemical exposure occurs as a response to a mixture rather than to a single chemical. Thus, evaluation of single chemical toxicity for specific species and environmental compartments may not show the real toxicity data in real life [1]. However, the assessment of a mixture’s toxicity is much more complex than toxicity evaluation of a single chemical. Interactions of chemicals in a mixture can reason for complex and significant changes in the apparent properties of its components. The components in a mixture may show ideal additive behavior of response/effects or may induce either increased (synergistic) or decreased (antagonistic) effects. Another problem is the identification of all existing components and their concentration in a specific mixture. In most of the cases, concentration of a chemical present is far below its individual median effective concentration 50% (EC_50_), some times even below its individual, no observed effect concentration (NOEC). The problem is that these compounds can still alter substantially the toxic effect of the chemical which share the majority of the mixture concentration. Thus, toxicity checking of just this major component may not show the real toxicity value for the final mixture [11,12].

**Figure 1:**
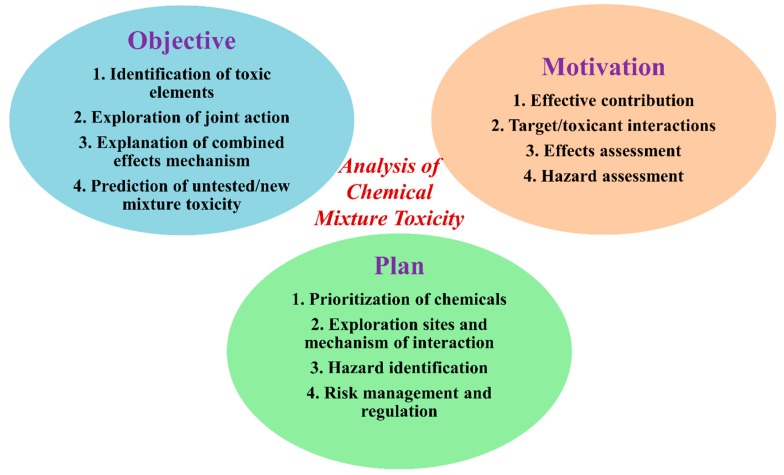
The objective, motivation and plans behind the analysis of a mixture’s toxicity.

Chemical regulation, acts and laws require the toxicity data for mixtures for risk assessment. The majority of regulatory authorities consider experimental as well computational toxicity data for mixtures to prepare risk assessment guidelines and awareness programs in public health all over the world. The most common acts that consider chemical toxicity data in the USA are the Federal Food, Drug and Cosmetic Act Federal Insecticide, Fungicide and Rodenticide Act, Food Quality Protection Act, Clean Water Act, Clean Air Act, Toxic Substances Control Act, Safe Drinking Water Act, Health Act, Occupational Safety, etc. [13]. In Figure 1, we summarize the major goal, motivation and outcome from the evaluation to illustrate the different objectives, foci, and intentions in joint action analyses of mixtures.

## 3. Hypothesis for a Mixture’s Toxicity Exploration and Data for Computational Modeling

An approach that is valid for toxicity assessment of single chemicals may not be relevant for mixtures as multiple complexities exist in the evaluation of their toxicity. In the case of pharmaceuticals, dose-response models for mixtures differ conditionally according to the dose ratios of components in the mixture. On the other hand, in the case of chemical mixtures existing in the environment, one needs to check the probable mixture concentration ratio of individual chemicals. A combination of two or multiple chemicals may modify the properties of an individual chemical and can create completely different physicochemical features which as a result affect the toxicity response of the final mixture. Based on the literature, chemical mixtures show joint action which is the most common and acceptable hypothesis among scientists. It is important to remember that the assessment of joint action necessitates distinctive types of pharmacological interactions. Bliss classified the joint action of mixtures into three distinctive categories [14]:

(i) If chemicals in a mixture showed same mechanism of action for a specific response and act on same site of action, then there are chances of dilution of the response. This method is known as concentration addition (CA).
(ii) If chemicals in mixtures act on different sites of action with dissimilar modes of action (MOA), this may disclose statistically independent responses without interaction. This method is known as independent action (IA).
(iii) If chemicals are interactive in nature, then they may show synergistic or antagonistic effects.

Most of the existing modelers consider that CA and IA are two popular approaches for evaluation of mixture toxicity under the joint action hypothesis. To prepare the toxicity data for modeling purposes, one needs to follow the steps mentioned below: 

### 3.1. Determination of Dosage Response Curves for All Chemicals in a Mixture

A dosage response (DR) curve should be generated for each chemical by employing a model organism with different concentrations of the respective chemicals. 

### 3.2. Determining the Effect of the Chemical Mixture

The investigator should then experimentally evaluate the effect of a chemical mixture to the model organism with and without the mixture. Here, one should not perform only one point experiment for the mixture but should measure dilution series of the mixture which will allow a complete dose response curve of the mixture to be attained. 

### 3.3. Modeling with Identified Hypothesis

#### 3.3.1. Concentration Addition (CA)

As mentioned earlier, the CA model assumes that compounds act via a similar mechanism to produce an effect, and thus one chemical acts as a dilution of the other and can be replaced at a persistent quantity for the other [15]. The CA model can be explained by the Loewe additivity equation. For instance, the equation will be the following for the binary mixture of compounds 1 and 2:

(1) C1ECy1+C2ECy2=1

where *C*_1_ and *C*_2_ are the specific concentrations of the compounds 1 and 2 creating the mixture, which results in an effect *y*, and *EC_y_*_1_ and *EC_y_*_2_ signifying the corresponding effect concentrations of the solo compounds 1 and 2 that alone would generate the same response *y* as the mixture. The combined effect or sum of *c*_1_ and *c*_2_ is *y*. Interestingly, the sum of Equation (1) is always equal to 1 for the CA modeling.

#### 3.3.2. Independent Action (IA)

Compounds act independently and have dissimilar MOA. The collective effect is computed employing the effects of components and their interactions [15]. The IA modeling can be explained through the following formula:

(2) $$E=1-(\left(1-e_{A}\right)(1-e_{B})(\ldots ))$$

*E* is the outcome of the mixture at an explicit concentration; *e_A_* is the effect of compound A at that definite concentration and *e_B_* is the same for chemical B. The equation can be expanded from binary mixtures to mixtures of more components.

#### 3.3.3. Synergistic and Antagonistic Actions

The toxicity of synergistic action is superior to that of components, while the antagonistic act has lower toxicity than that of the components. Considering the Loewe additivity equation (Equation (1)), when the sum is higher than 1 (>1) then this suggests that a higher total concentration is required to produce the same effect which assumes an antagonistic effect (infra-additive). If the value is lower than 1 (<1), then it is a synergistic effect (supra-additive) [15].

#### 3.3.4. Generalized Concentration Addition (GCA) Models

The CA and IA models are unsuccessful for chemicals that have high potency but low efficacy. Thus, a generalized concentration addition (GCA) model was created by Howard and Webster to eliminate these limitations [16]. The GCA considers the cumulative effect of a mixture by means of the efficacy and potency of the mixture’s constituents. The GCA model can be explained by the following equation [17]:

(3) E=max effect level A[A]EC50A+max effect level B[B]EC50B+…1+[A]EC50A+[B]EC50B+…

*E* is the effect of the mixture at a definite concentration. Whereas, ‘max effect level A’ is the maximal effect level of compound A, [*A*] is the concentration of A in the mixture at an explicit mixture concentration, *EC*_50*A*_ is the *EC*_50_ value of A and similar for chemical B, etc.

## 4. Importance of Computational Approaches to Determine the Toxicity of Chemical Mixtures

**Figure 2:**
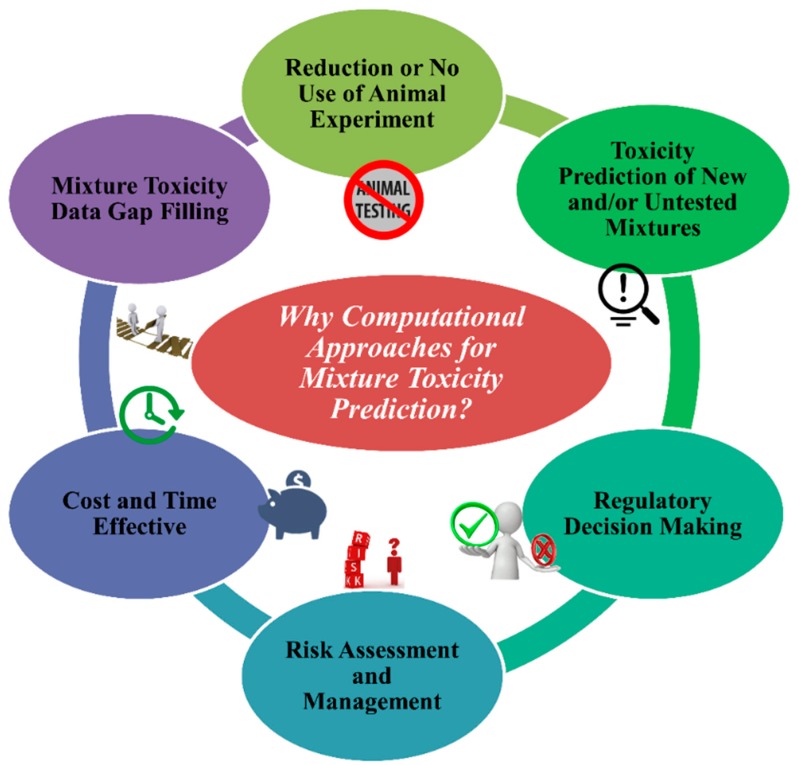
The reasons behind the implication of computational approaches for a mixture’s toxicity prediction.

A huge number of single chemicals already existing in the system have no toxicity data for living systems as well as for the environment. The scenario for mixture toxicity data is even worse. To evaluate the toxicity for single compounds and mixtures employing animal models can take many years with the expense of billions of dollars [18]. In silico methods are one of the promising approaches for toxicity assessment employing multiple algorithms and expert systems that use computation [19]. It is important to mention that computational approaches are not the absolute alternative or substitution for in vivo and in vitro toxicity tests. Rather, they aim to complement experiments by minimizing animal testing, decreasing the cost and time of toxicity tests, followed by advanced and improved toxicity prediction and risk assessment. Additionally, in silico methods can in advance predict the toxicity of mixtures with any combination. The major significance (Figure 2) of computational approaches for toxicity prediction of chemical mixtures is the following:

(1.) To stop the unethical use of animal cruelty in the name of animal modeling. The application and acceptance of in silico approaches can decrease the use of animals in toxicity testing.
(2.) Using in silico models from existing chemical mixtures, one can assess/predict the toxicity of untested and/or new different combinations of chemical mixtures for a specific species or systems if they fall under the applicability domain (AD).
(3.) Regulatory agencies like United States Environmental Protection Agency (US EPA), European Union regulations like the Registration, Evaluation, Authorization and Restriction of Chemicals (REACH), and Health Canada consider and depend on in silico methods for toxicity and risk assessment followed by decision making.
(4.) In silico methods are reliable tools to analyse the quantity of risk followed by methods to manage it.
(5.) Without any doubt, in silico tools are cost- and time-effective compared to in vivo and in vitro methods.
(6.) A reliable source of methods to fill gaps in mixture toxicity data as the majority of mixtures have no toxicity data at all.

## 5. Types of Computational Approach for a Mixture’s Toxicity Prediction 

**Figure 3:**
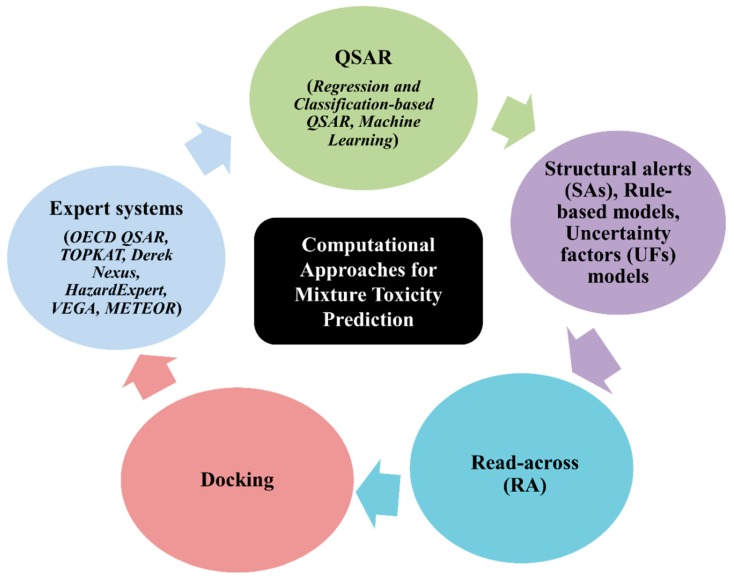
Types of computational approaches for the prediction of toxicity.

Since the introduction of computational approaches, a good number of in silico models have been established to predict the toxicity of chemicals. In the present review, we reveal the most common types of such methods that are applicable for a mixture’s toxicity prediction only (Figure 3). Extensive details can be found elsewhere [20,21,22,23,24] as in the present review we intend to place more emphasis on a mixture’s toxicity prediction hypothesis.

QSAR: the quantitative structure-activity relationship (QSAR) methodologies are the most commonly employed computational approaches that correlate the toxicity of a compound and its structural features [21,22]. A QSAR model is developed by engaging a series of chemicals with a certain response, here, toxicity. It helps in screening the toxicity of large databases of untested and/or new molecules bearing the specific response. The QSAR technique regulates the structural attributes of the molecules accountable for their toxicity. The QSAR analysis is based on the notion that toxicity (T) depends on the structure (C) of a studied chemical.

(4) T=f(C)

Then, the chemical structure can be represented as descriptors to correlate the toxicity in the case of QSAR modeling. Therefore, the ‘*C*’ notation can be substituted with the term ‘Descriptors’. Descriptors illustrate specific information from the chemical structure itself along with its inherent property in the form of numerical value. 

In the beginning, molecular properties and constitutional indices are considered as descriptors (OD and 1D form of descriptors). Along with the advancement of the theoretical graph, the importance of 2D descriptors arises. During the late 1980s, a molecular interaction-based feature between the compound and a probe chemical was introduced to mark the era of 3D QSAR. Afterwards, multidimensional QSAR techniques flourished e.g., 4D-QSAR, 5D-QSAR, 6D-QSAR and 7D-QSAR. Thus, based on dimensionality, QSAR can be classified from 0D to 7D (Table 1).

**Figure 4:**
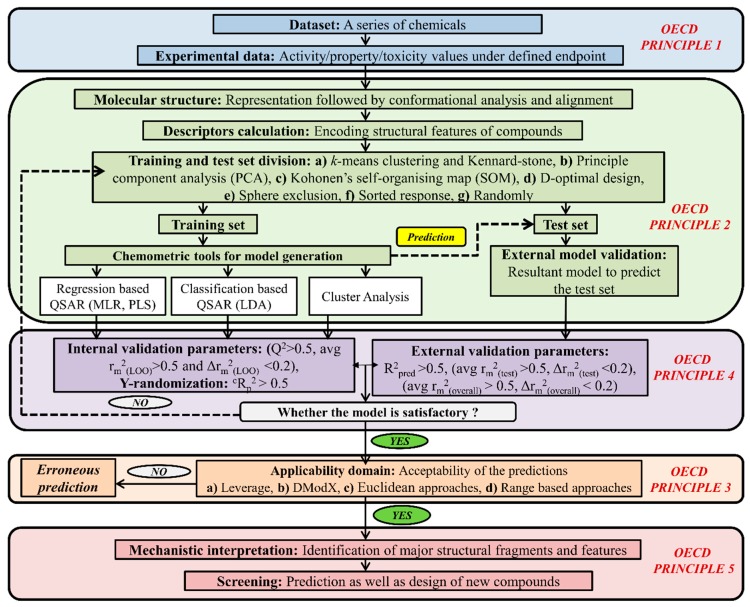
A complete schematic representation of the development of a QSAR model.

On the other hand, based on chemometric tools, it can be classified as regression-based QSAR (partial least squares (PLS), multiple linear regression (MLR), genetic function approximation (GFA), genetic partial least square analysis (G/PLS)), classification-based QSAR (linear discriminant analysis (LDA) and cluster analysis (CA). Machine learning methods like the support vector machine (SVM), artificial neural network (ANN), random forest (RF) are also an important tool to develop models. The QSAR model has the capability to identify the responsible structural features as well as the physical property for toxicity along with the exploration of a possible mechanism behind the toxicity of a specific class of chemicals to definite species. How QSAR models are built and validated is demonstrated in Figure 4. 

Structural alerts and rule-based models: structural alerts (SAs) can be considered as responsible structural fragments or toxicophore associates to toxicity. SAs can be from single atoms to a large fragment as well a combination of fragments. Again, single SA or multiple SAs can contribute to toxicity [31,32]. On the other hand, generally two types of rule-based models exist: induction-based rules (IBRs) and human-based rules (HBRs) [31]. IBRs can be created competently from big datasets. IBRs recommend theories about relations between a molecule’s structural and toxicity endpoints which may not be explored from human insights [32]. By contrast, HBRs result from human knowledge of experts whereas IBRs are derived computationally. As HBRs largely depend on human knowledge, that is why chances of bias are high. Based on the requirements and case, a hybrid-based rules system which is a combination of IBRs and HBRs can be generated. 

The advantage of SA and rule-based methods:

(1) Methods are easy to implement and interpret.
(2) Help to determine how compounds should be transformed to decrease their toxicity.
(3) Capable of categorizing the structure of likely metabolites.

Disadvantage of SA and rule-based methods:

(1) The presence or absence of SAs does not offer understanding of the biological pathways of toxicity.
(2) If all SAs are not identified properly, the method can increase false negatives.

Read-across: read-across (RA) is a tool for predicting the untested toxicity of a chemical using chemical analogs with existing experiemental toxicity from the identical chemical category [33]. In the case of read-across, endpoint information for the source chemical is employed to predict the same endpoint for the target chemical, which is considered to be “analogous” on the basis of structural similarity or mechanisms of action. The RA approach is helping to fill the data gaps under REACH and the US EPA for thousands of chemicals, being very fast for comparing experimental and animal models. The RA approach can be classified into two types: the analog approach (AN) which is defined as one-to one uses of one or few analogs, and a category approach (CA) which signifies many-to-one uses of multiple analogs. The AN method is sensitive to outliers as two analogs may have dissimilar toxicity profiles. On the other hand, the CA is beneficial for noticing trends within a category and may raise confidence in the toxicity predictions.

Docking: molecular docking is an approach focusing on the fitting of two molecular structures, for example small molecules (chemicals, pharmaceuticals) with large molecules like enzymes or receptors. The capability of interaction between small molecule and large molecules forming a supra-molecular complex serves a significant role in governing the required biological activity. The objective of the in silico method is to recognize the exact poses or orientation of ligands (small molecule) in a binding pocket of a protein (large molecule) and to predict the affinity between ligand and protein. It can be categorized as: (i) drug molecule–protein docking, (ii) nucleic acid–protein docking, and (iii) protein–protein docking. The precision and quality of a docking relies on the search algorithm (genetic algorithms, Monte Carlo methods, Tabu searches, fragment-based methods) and scoring functions (empirical free energy scoring functions, force-field methods) [21,22].

**Figure 5:**
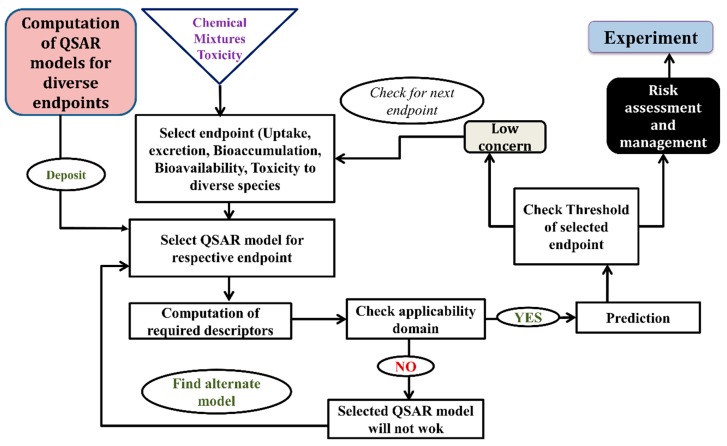
Flow chart to prepare QSAR-based expert system.

Expert systems: expert systems are convenient choices for the prediction of toxicity over the old-style and/or local QSAR models as they require only structure, endpoint and environment compartment as an input. Expert systems can be defined as a combination of multiple QSAR models and/or QSAR models along with other computational approaches like RA, SA, IBR etc. in a form of user-friendly system or software. For fast and cost-effective prediction followed by risk assessment, and regulatory guidelines’ generation, expert systems are highly effective and can be handled by non-experts also. Expert systems may offer widespread structural and mechanistic intricacy regions compared to the local QSAR models. Most promising expert systems for toxicity predictions are the following: AMBIT, AIM, Toxtree, OCED Tool Box, DSSTox, Derek Nexus, Meteor, CASE, HazardExpert, PASS, cat-SAR, Toxmatch, AmbitDiscovery, VEGA, ChemIDplus. The details of each expert system can be found elsewhere [34]. Employing the QSAR equation, a computational chemist can build their own expert system using the following flowchart reported in Figure 5.

## 6. Successful Application of Computational Modeling for Predicting a Mixture’s Toxicity

There have been successful applications for prediction of toxicity of mixtures using computational methods. Tichý et al. [35] developed QSAR models for acute toxicity of aqueous solutions of binary mixtures of inorganic salts where toxicity was determined as 50% inhibition of movement of *Tubifex tubifex* worms. Authors considered interspecies differences, dynamics of the effect, numerous parameters of the same species or inter-individual differences, etc. to encode the toxicity index for mixtures. The study described an algorithm involving a test of additivity and generation of a mathematical explanation of the relationship between the index of acute toxicity EC_50_ of the mixtures and composition of binary mixtures. Agreement between the calculated and measured toxicity data can be expressed with the correlation value of 0.936 for 22 datapoints. Authors explained that the molar ratio R is the most suitable descriptor of the mixture composition that can be determined experimentally. They developed a mathematical equation with polynomial function describing the dependence of EC_50_ on R that was demonstrated to be beneficial for the presented study. The molar ratio, R, was computed as shown by Equation (5):

(5) R=xA(xA+xB)

where *x_A_* and *x_B_* are corresponding molar concentrations of binary mixture components A and B. The most appropriate polynomial is suggested in the form:

(6) $$EC_{50j}=1+a_{1}R_{j}+a_{2}R_{j}^{2}+a_{3}R_{j}^{3}+a_{4}R_{j}^{4}+a_{5}R_{j}^{5}$$

where, *R_j_* and *EC*_50*j*_ are the molar ratios of the j^th^ binary mixture and normalized values of the acute toxicity. The constants a, (n = I - 5) are regression coefficients calculated as shown above.

**Figure 6:**
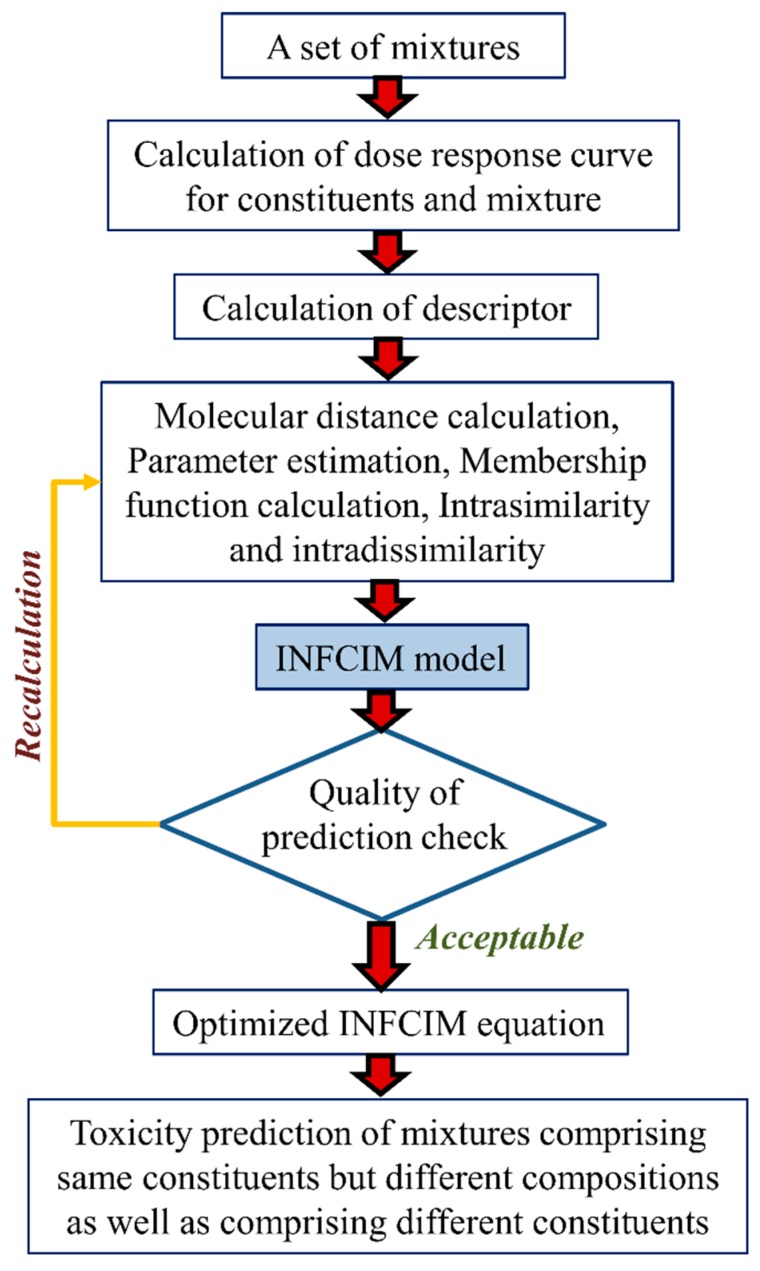
Strategy behind the integrated fuzzy concentration addition-independent action model (INFCIM).

Mwense et al. [36] proposed a methodology named the integrated fuzzy concentration addition-independent action modeling (INFCIM) approach which employs fuzzy set theory and molecular descriptors to illustrate the degree of likeness and differences of mixture components, and integrates the independent action and concentration addition models (Figure 6). Authors tested their approach in two case studies employing four mixtures’ datasets and most importantly, the obtained results were compared with those of both independent action and concentration addition models. The first mixture dataset comprises 18 *s*-triazines acting on green freshwater algae *Scenedemus* *vacuolatus* whereas the second dataset consists of 16 acting chemicals tested on *Scenedemus* *vacuolatus*. Mixture 3 and 4 datasets comprise 10 quinolone compounds and 16 phenol-derivative compounds causing long-term inhibition of bioluminescence in the bacterium *Vibrio fischeri*. The proposed INFCIM approach can be considered a QSAR like tool for mixtures’ toxicity prediction. The method employs an equation with only two parameters that it is essential to check for the fuzzy membership functions and can be evaluated using only one set of mixture data, i.e., the concentration response curve of the mixture for a given composition. Authors claimed that the INFCIM model can be used to predict toxicity of the mixture at any composition. The prediction errors between the experimental results and INFCIM was less than 10% for the s-triazines dataset, within 16% for second dataset, approximately 11% for both the quinolone and phenol derivatives’ mixture datasets. The results obtained suggested that INFCIM performs comparably to or improves on the best acting current model for all the mixtures tested.

Boeijea et al. [37] reported QSAR models applied to two binary mixtures of alcohol ethoxylate (AE) ecotoxicity for fish, invertebrates, and mesocosms. Authors suggested that their models are better than the existing models considering statistical accuracy and reliability. Most importantly, the developed models showed a correlation coefficient of 95% and higher for all three ecotoxicological endpoints, i.e., *Daphnia magna*, *Pimephales promelas* and mesocosms. In the case of ethoxymer distributions of commercial AEs, the developed QSAR model predicts less toxicity than the QSARs based on an average structure. Not only that, the models are also appropriate for the prediction of the ecotoxicity of sole components or, via toxic units addition, of environmental fingerprints. Boeijea et al. [37] recommended following chronic QSARs for calculating, and predicted no effect concentration (PNEC) derivation in environmental effects valuations:

Daphnia *EC*_20_ QSAR:
  (7) $$EC_{20}\;=10^{-0.532\times log\;K_{ow}+2.975}\;(\mu mol/L)$$
Mesocosm NOEC QSAR:
  (8) $$EC_{20}\;=10^{-0.740\times log\;K_{ow}+3.22}\;(\mu mol/L)$$

The QSAR model for toxicity of 50 binary mixtures to *Photobacterium phosphoreum* (T3 mutation) had been developed by Toropova et al. [7] where toxicity is expressed as effective concentration essential to a 50% decrease in light emission. To model the toxicity, authors computed simplified molecular input-line entry system (SMILES)-based descriptors employing the Monte Carlo optimization approach. The QSAR models were developed based on six different splits consisting of sub-training, calibration, and test sets without including the validation set. The models showed following statistical results: N = 38, R^2^ = 0.95, s = 0.16 (split 1); N = 39, R^2^ = 0.93, s = 0.19 (split 2); N = 37, R^2^ = 0.92, s = 0.22 (split 3); N = 33, R^2^ = 0.94, s = 0.20 (split 4); N = 36, R^2^ = 0.89, s = 0.24 (split 5); N = 39, R^2^ = 0.94, s = 0.18 (split 6). Based on mechanistic interpretation, authors concluded that the presence of bromine, chlorine and oxygen is the promoter of toxicity enhancement. By contrast, the nitrogen helped in decreasing the studied toxicity.

Tian et al. [38] developed a QSAR model to predict joint effects at non-equitoxic ratios for binary mixtures consisting of cyanogenic chemicals, reactive toxicants, and aldehydes. The obtained result from the study demonstrated that the relationships between toxic ratios of the specific chemicals and their joint effects can be designated by a normal distribution function. Authors suggested that based on normal distribution equations, the joint effects of binary mixtures at non-equitoxic ratios $\left(TU_{sum}^{n:m}\right)$ can be predicted quantitatively using the joint effects at equitoxic ratios $\left(TU_{sum}^{1:1}\right)$. The developed QSAR model can predict the joint effects of mixtures at non-equitoxic ratios $\left(TU_{sum}^{n:m}\right)$. It has been validated employing external mixtures other than the modeled ones. The noteworthy correlation between the experimental and predicted results (R = 0.941) specifies that the predicted outcomes of joint effects for mixtures are reliable with the observed results at non-equitoxic ratios. The reported study offers a method for the prediction of joint effects for binary mixtures at non-equitoxic ratios.

Mo et al. [39] employed mixtures of 22 phenolic and aniline derivatives (PADs) to investigate if the dose addition and independent action models can be used to evaluate their toxicity. A mixture’s photobacterium toxicity to the *Vibrio qinghaiensis* sp. Q67 showed that the two-parameter Logit function or Weibull approach could be efficiently useful for defining the dose-response relationships. with *R* > 0.99 and root mean squared error (RMSE) < 0.037, which illustrated the stability and calibration capability of the fitting of the two models. Authors concluded that the joint toxicity of 12 uniform design concentration ratio (UDCR) mixtures and three equivalent-effect concentration ratio (EECR) mixtures could be assessed well by means of the dose addition (DA) or the independent action (IA) model within 95% confidence intervals. Authors predicted the dose response curves (DRCs) based on both of the DA and IA models employing the APTox program. The DA model can be expressed through the following equation:

(9) $$\sum_{i=1}^{n}\frac{c_{i}}{EC_{x,i}}=1$$

where *c_i_* the concentration of the *i*^th^ component in the mixture, *EC_x,i_* is the concentration of the *i*^th^ component that provokes *x*% effect when applied singly, and *n* is the number of mixture components. Again, the IA model can be mathematically expressed as the following:

(10) $$E(c_{\mathrm{mix}})=1-\prod_{i=1}^{n}\left(1-E(c_{i})\right)$$

where *E*(*c*_mix_) and *c*_mix_ are total effect of the mixture and the total concentration, respectively, and *E*(*c_i_*) is the effect of the *i*^th^ component with the concentration of *c_i_* in the mixture.

Wang et al. [40] developed classical QSAR models to estimate the toxicity of 99 binary mixtures of organic chemicals. The QSAR models were generated employing non-linear radial basis function neural networks (RBFNNs) and forward stepwise multiple linear regression (MLR) utilizing the hypothetical descriptors. The hypothetical descriptors for the modeling of mixtures resultant from the descriptors (QC Max, NTB and ACIC2) identified from the model developed with single chemicals were effectively utilized to quantity the contributions of the element of a mixture to the toxicity. The statistical qualities of the MLR model provided were *R*^2^ = 0.869 and *Q*^2^_LOO_ = 0.864 for the training set, and *R*^2^ = 0.853 and *Q*^2^_ext_ = 0.825 for the test set. The RBFNN model gave the statistical parameters *R*^2^ = 0.925 and *Q*^2^_LOO_ = 0.924 for the training set, and *R*^2^ = 0.896 and *Q*^2^_ext_ = 0.890 for the external test set. The statistical results are very acceptable and the residuals between experimental and predicted toxicity for the majority of the mixtures are within the 5% range. Based on the presented result, the author found out that MLR can predict the mixture toxicity more precisely than the RBFNN model.

Kar et al. [41] developed QSAR models employing toxicity data on zebrafish embryos of 9 halogenated chemicals contain 5 single (TBBPA, TDCPP, PFOA, DOPO, and PFBA) and 4 tertiary mixtures. The QSAR model was developed employing genetic function algorithm tool using weighted descriptors approach. The models can express regression correlation range from 0.73–0.87 and while it could predict 54%–65% of the variance (leave-one-out predicted variance). Considering the test set, the models showed a correlation range from 0.60 to 0.73. The developed model was further employed by authors for toxicity prediction of 2,340 compounds consisting of single, binary and tertiary halogenated mixtures as well as perfluoroalkyl substances (PFASs). The developed model suggested that the studied chemicals in mixtures exhibited concentration addition (dose addition) of individual chemicals which account for similar MOA and non-interaction of chemicals. Additionally, mixtures of halogenated compounds including PFASs displayed the following toxicity trend: single chemical > binary mixture > tertiary mixture.

Quin et al. [42] had developed a QSAR model for the acute toxicities toward *Aliivibrio fischeri* of 45 binary and multi-component mixtures consisting of four pesticides and two antibiotics. A genetic algorithm (GA) tool was employed to attain the three theoretical descriptors’ model with acceptable internal (*R*^2^ = 0.94, *Q*^2^_LOO_ = 0.91) and external (*R*^2^_pred_ = 0.78) validation parameters. The three modeled descriptors identified by GA are RDF035m (specifies the probability distribution of finding an atom in a spherical volume of radius R), HATSs (designates leverage-weighted total autocorrelation index/weighted by intrinsic state), and H-047 (defines that H^a^ is attached to C^1^(sp3)/C^0^(sp2), where ‘a’ signifies the formal oxidation number). The obtained model presented more precise additive, antagonistic and synergistic toxicities of mixtures compared with traditional CA and IA models. Thus, the QSAR model may be employed to predict the non-additive and additive toxicities of mixtures.

**Figure 7:**
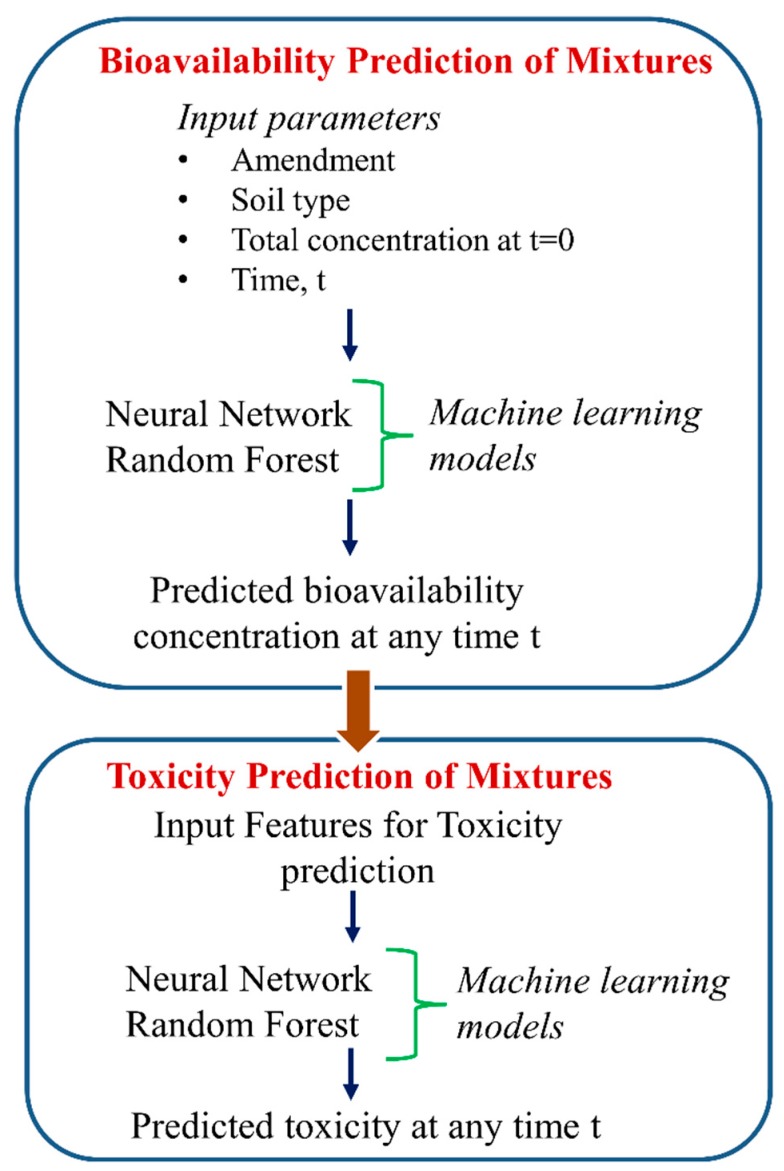
Prediction of soil toxicity by two-stage approach employing bioavailability.

Cipullo et al. [43] employed two machine learning (ML) models, including random forest (RF) and artificial neural networks (NN) to predict temporal bioavailability followed by toxicity prediction employing predicted bioavailability features as the input of complex chemical mixtures (Figure 7). The authors employed 6 months of mesocosms experimental data to analyze total and bioavailable heavy metals/metalloids content and petroleum hydrocarbons. Features like amendment (biochar and compost), soil type, initial concentration of specific chemicals, and incubation time were employed by authors as inputs of the ML models to better understand the drivers of temporal changes in bioavailability and toxicity. The developed ML models expressed that each toxicity response is dependent on different features. Results showed that the prediction of the earthworm acute toxicity index was mainly driven by acenapthene (AE), anthracene (A), phenantrene (P), fluorene (F), pyrene (PY) and EC_17_–EC_19_ (aliphatic fraction 17 to 19). The aliphatic compounds with mid-chain length were identified as a vital indicator of acute toxicity to soil organisms like earthworms. Additionally, this fraction combined with small aromatic compounds showed more bioavailability with the highest toxicity potential (for example: phenanthrene and acenaphtene). Authors suggested that the toxicity of mixtures may not be correctly predicted using classical regression analysis rather than multiple factors (combined effects)-based analysis accounts for correct prediction. The implication of ML models could advance the understanding of rate-limiting processes disturbing the spontaneously accessible fraction of pollutants in soil followed by a contribution to the mitigation of potential risks. The study strengthens the idea that the bioavailability of multiple metals and hydrocarbons drives the soil toxicity and ML models can be a fast and economic option to monitor multi-contaminated sites.

## 7. Future Avenues of Chemical Mixture Toxicity Research

The idea of chemical interactions in a mixture is not new, but the requirement of mixtures’ toxicity assessment and awareness evolved much later than the analogous concerns for single chemical toxicity assessment. The idea and necessity are understood by all regulatory authorities and it is clear for toxicologist that to obtain a complete picture of toxicity assessment one needs to check mixture toxicity data rather than focus on a single chemical. The ATSDR developed a strict direction for chemical mixtures which is equally like that in the U.S. EPA guidance, although the ATSDR offers more weighting on physiologically based pharmacokinetic (PBPK) and pharmacodynamic (PBPD) modeling. Agencies like the NIEHS, National Toxicology Program (NTP) and National Institute for Occupational Safety and Health (NIOSH) initiated efforts to illustrate exposures, generate biomarkers, and assess environmentally relevant mixtures [44].

The database with most of the toxicity information followed by an experimental protocol is very important to generate computational modeling followed by expert systems. These expert systems may finally be beneficial for mixtures’ assessment or, at the very least, for predicting dose-dependent interactive effects. In the present time, a hand-countable database covers the mixture toxicity data. Thus, a large number of efforts needs to be employed to prepare an improved database in collaboration with an experimental and computational modeler. Another significant future introspection requires in the field of validation of the expert systems which are responsible for toxicity data gap filling of mixtures. If expert systems are not properly validated and not universal enough, then the chances of error will be significant. A significant amount of attention is also required to the applicability domain aspect as one cannot use any expert system to predict a new and/or untested mixture’s toxicity.

Considering present and future aspects of the mixture toxicity study, we recommend the following points for future efforts:

(a) Mixture assessment should use low doses, for example up to the no-observed-adverse-effect level (NOAEL);
(b) There is no ultimate or universal method, and one needs to develop new or modified approaches from case to case to address the complex issue of mixture, noting that old-style animal-based toxicology practices are insufficient for such a multifaceted issue;
(c) Collaborative efforts between experimentalist and computational communities are must to address majority of issues and challenges related to mixture toxicity;
(d) A variant of the Hausdorff measure, called Hausdorff-like similarity (Hs), can be useful in modeling a complex system like mixtures [45]. To quantify the similarity degree between two systems, it is not suitable to account only for mutual or dissimilar features, but all the features of the systems have to be measured in the assessment. Hausdorff formula are capable of equally weighing both the existence of common/comparable elements. To measure the diversity relationship between the two sets X and Y, the Hausdorff formula can be defined as follows:
  (11) $$dHaus_{XY}=max\left\{\begin{array}{c} sup\\ x\in X \end{array}\left[\begin{array}{c} inf\\ y\in Y \end{array}\left(d_{xy}\right)\right],\begin{array}{c} sup\\ y\in Y \end{array}\left[\begin{array}{c} inf\\ x\in X \end{array}\left(d_{yx}\right)\right]\right\}$$
  from which the equivalent similarity measure can be calculated as:
  (12) $$sHaus_{XY}=min\left\{\begin{array}{c} sup\\ x\in X \end{array}\left[\begin{array}{c} inf\\ y\in Y \end{array}\left(s_{xy}\right)\right],\begin{array}{c} sup\\ y\in Y \end{array}\left[\begin{array}{c} inf\\ x\in X \end{array}\left(s_{yx}\right)\right]\right\}$$
  where the signs *s* and *d* denote the similarity and the distance measures, correspondingly.

The Hausdorff-like similarity can be defined as following between the two sets *X* and *Y*:

(13) $$Hs_{XY}=\frac{\sum_{x\in X}\begin{array}{cc} \begin{array}{c} max\\ y\in Y \end{array} & \left[s_{xy}\right]+\sum_{y\in Y}\begin{array}{cc} \begin{array}{c} max\\ x\in X \end{array} & \left[s_{yx}\right] \end{array} \end{array}}{n_{X}+n_{Y}}$$

where, *s_xy_* and *s_yx_* are any pair-wise similarity measures between the *p*-dimensional elements *x* and *y* of the sets *X* and *Y*, respectively. The terms under the numerator signify the maximum similarity between the individual element for both sets; *n_X_* and *n_Y_* are the number of elements for both sets.

## 8. Conclusions

The most significant role of in silico methods for chemical mixtures is the assessment and prediction of influences of these complex substances to the human system as well as the environment. Most of the time, scientists perform risk assessment through toxicity investigating single chemicals but the fact is that most chemicals exist as mixtures, mostly at very low levels of the discrete concentrations. Thus, the risk assessment may be misleading in the majority of cases. The identification of each chemical present in the mixture as well as their combination ratio is very important before performing any toxicity quantification. Although all the facts are known to environmental and toxicity scientists, the availability of a mixture’s toxicity data is really scarce. The requirement of a toxicity study of a mixture needed in the present era without any doubt and the computational approach is the answer that assists in completing the mixture toxicity data economically, with minimum time. The present review demonstrates the hypothesis behind mixture toxicity modeling, available in silico tools and/or computational approaches. We discussed and revealed a successful application of in silico tools for toxicity assessment models. In our expert opinion, we can suggest that the identification of each component and their mechanism of action is necessary behind toxicity, followed by modeling considering the identified mechanism. Another important point to remember, considering physicochemical parameters related to the mechanism of action, is that the developed model can replicate the toxicity response in a mathematical equation. Such an equation could be used for predictions for untested compounds/mixtures.

## Figures and Tables

**Table 1:** Classification of quantitative structure-activity relationship (QSAR) analysis based on dimension.

Dimension	Description	Representative Example of Descriptors or Computational Method	Reference
0D	Chemical formula derived descriptors	Constitutional indices (Molecular Weight (MW), sum of properties etc.), molecular property descriptors, count descriptors (count of bond, atom, non-hydrogen atom etc.)	[25]
1D	Descriptors are derived using the representation of various sub-structural molecular fragments	Fingerprints, count of fragments, H-Bond acceptor/donor, Crippen AlogP98, PSA, SMARTS etc.	[25]
2D	Descriptors are obtained from the graph theoretical representation of molecules including various structural and/or physicochemical property indices	Topological descriptors, eigenvalue-based descriptors, connectivity indices, descriptors containing topological and electronic information.	[25]
3D	These independent variables encode various spatial as well as geometrical information of compounds and are derived using 3D representation of structure. Such parameters basically portray static representation of a ligand.	WHIM descriptors, MoRSE descriptors, Jurs parameters, GETAWAY descriptors, quantum-chemical descriptors, atomic coordinates, size, steric, surface and volume descriptors. Techniques e.g., Comparative Molecular Field Analysis (CoMFA), Comparative molecular similarity index analysis (CoMSIA) etc.	[25,26]
4D	Depict multiple representation of the ligand molecule using various configurations, orientation, and protonation state representation.	Volsurf, GRID, Raptor etc. derived descriptors.	[27]
5D	Descriptors consider the induced fit parameters and aim to establish a ligand-based virtual or pseudo receptor model.	Flexible-protein docking.	[28]
6D	These are derived using the representation of various solvation circumstances along with the information obtained from 5D-descriptors.	Quasar.	[29]
7D	Such analysis comprises real receptor or target-based receptor model data.	−	[30]
